# Patient Preference for Once-Weekly Dosing in Type 2 Diabetes Mellitus in Japan

**DOI:** 10.36469/9826

**Published:** 2016-03-18

**Authors:** Rohini Sen, Alan L. Shields, Koichiro Atsuda

**Affiliations:** 1 Adelphi Values, Boston, MA, USA; 2 Kitasato University, Kanagawa, Japan

**Keywords:** patient survey, weekly and daily dosing, patient preferences, diabetes treatment, type 2 diabetes mellitus

## Abstract

**Background:** Among several factors that impair adherence to available therapies in type 2 diabetes mellitus (T2DM) is the complexity of the dosing regimen. Moreover, the value of a once-weekly (QW) administration of oral medications for T2DM compared to once, twice, or thrice daily (QD, BID, TID) regimens is unclear. This study aims to identify subgroups and patient characteristics correlated with a preference for QW dosing compared to daily dosing using survey-based methods.

**Methods:** This was a cross-sectional online survey study among patients with T2DM in Japan. Patients with T2DM were categorized into one of the three groups: (1) patients on treatment with oral hypoglycemic agent(s) only, (2) patients on combination treatment with oral hypoglycemic agent(s) and insulin, and (3) patients diagnosed with or suspected to have T2DM with no current or past experience with T2DM drug treatment (treatment naïve). Preliminary logistic regressions and classification and regression tree analysis (QW/QD dosing preferences as the dependent variable) were conducted to identify key predictors of dosing preference, followed by an evaluation of frequencies and trends in dosing preferences by the identified factors (subgroups).

**Results:** Current treatment regimen, age, and work status were identified as the major demographic factors that were most predictive of QW preference. While, overall, 55.5% preferred QD and 33.3% preferred QW, the preference toward QW is higher in a specific cohort of patients that is treatment naïve (i.e., patients diagnosed with T2DM and/on diet/exercise therapy with no current or past experience with T2DM drug treatment) than who are on treatment, younger (age ≤64 years old), working full-time than part-time, and/or currently taking 0 or 1 drugs or more than 6 drugs (68.67% versus 30.12%). The most commonly cited reasons for QW preference were (1) “less burdensome because they didn’t have to take it every day” (47.8%), (2) “less psychological burden” (14.6%), and (3) “forget to take it less often”(12.5%).

**Conclusion:** Patients with T2DM vary in terms of preference for dosing regimens. Daily dosing was preferred over QW dosing in the overall population, however, preference for QW was higher in younger, full-time working, treatment naïve subjects, who are/or currently taking 0 or 1 drugs or more than 6 drugs.

## INTRODUCTION

The International Diabetes Federation reported 7.2 million cases of diabetes and more than 64,000 related deaths among adults in Japan alone in 2014.[1] In addition to the human cost, diabetes imposes a tremendous burden on the Japanese health care system, with an estimated per-person health care cost of nearly $5,000 annually[1] – a cost that is poised to explode in the coming years.

As in much of the developed world, the incidence of diabetes has surged in Japan, rising by 29% in the decade from 1997 to 2007.[2] Owing to one of the lowest rates of type 1 diabetes (T1DM) in the world,[3] most of this growth has been driven by an increase in type 2 diabetes mellitus (T2DM), and like many chronic diseases, has occurred primarily among the elderly; the number of Japanese 60-79 years of age with diabetes is about double that of those aged 40-59 years.[4] Nevertheless, diabetes rates are rising among younger Japanese as well. More critically, these cohorts are less likely to be on treatment for their diabetes, and therefore represent a prime target for intervention.[5]

Poor adherence to available therapies is a major hurdle to the effective management of chronic diseases such as diabetes.[6,7] The World Health Organization estimates that long-term adherence rates in chronic diseases average only 50% in the developed world, the consequences of which include degraded health outcomes, impoverished quality of life, and increased health care costs.[6,8] Among the factors that have been shown to impair adherence is the complexity of the dosing regimen, including polypharmacy.[9-11] As patients with T2DM often have comorbid and co-occurring conditions, many receive multiple medications – as many as five or more medications daily.[12,13] As such, reducing complexity through moderated dosing frequency presents a compelling means of potentially improving adherence and, ultimately, health outcomes.[14] Indeed, reducing the frequency of dosing from twice to once daily (QD) has been shown to improve adherence in patients taking T2DM medication.[15,16]

Once weekly (QW) formulations of oral dipeptidyl peptidase-4 (DPP-4) inhibitors represent a significant departure from the existing treatment paradigm, characterized by daily, or more than once daily, dosing regimens. While such QW formulations have been approved for use in Japan, the value of QW administration of oral medications for T2DM compared to QD, twice daily (BID), or thrice daily (TID) drugs – amidst other, potentially inconsistent and overlapping regimens – is unclear.

The objective of the present study was to identify subgroups and patient characteristics correlated with a preference for QW dosing compared to daily dosing. By providing a patient-centric and evidence-based value demonstration of QW dosing, physicians may more readily identify those patients whom QW drugs will benefit.

## METHODS

### Study Design and Procedures

In an ethics board approved study, MACROMILL, a market research organization in Japan, identified and screened individuals through an online panel. A total of 15 833 individuals were approached with information regarding this study. Among the 12 842 panelists who responded, 6167 were eliminated due to the screening (inclusion/exclusion) criteria (Table 1), and 1409 agreed to participate. Among those who agreed to participate, 1134 (80.5%) completed/responded to the survey, a response rate consistent with or better than is typical in online survey research.[17,18] To qualify for participation, subjects had to be 40 years of age or older (a criteria to facilitate study enrollment in that T2DM patients are more likely to be at least 40 years of age),[27] self-report a T2DM diagnosis, and be on treatment with oral oral hypoglycemic agent(s) and insulin or have no current or past experience with treatment for T2DM (treatment naïve). Individuals with T1DM were excluded from the study. Once consent was obtained, the eligible subjects completed the survey using web-based methods and were compensated for their participation. Data were automatically and electronically downloaded into a comprehensive data set.

**Table 1.** Inclusion and Exclusion Criteria

| | Inclusion Criteria | Exclusion Criteria |
|---|---|---|
| Group 1 (n= 515)<br>T2DM and on treatment with OAD only | • Patients with diagnosed T2DM<br>• 40 years old or above<br>• Patients on treatment with OADs only | • Patients with diagnosed T1DM<br>• Patients on insulin treatment<br>• Patients only on diet/exercise therapy |
| Group 2 (n=258)<br>T2DM and on combination treatment with OAD and insulin | • Patients with diagnosed T2DM<br>• 40 years old or above<br>• Patients on combination treatment with OAD and insulin | • Patients with diagnosed T1DM<br>• Patients on oral treatment only<br>• Patients only on diet/exercise therapy |
| Group 3 (n=258)<br>Patient diagnosed with or suspected to have T2DM with no current or past experience with drug treatment for T2DM (treatment naïve) | • Patients with diagnosed T2DM (or pre-diabetes if too few diet/exercise subjects are recruited)<br>• 40 years old or above<br>• Patients with no current or past experience with treatment for T2DM | • Patients with diagnosed T1DM<br>• Patients on insulin or oral treatment<br>• Patients previously on oral or insulin treatment |

OAD: oral antidiabetic; T1DM: type 1 diabetes mellitus; T2DM: type 2 diabetes mellitus

### Study Sample

Table 2 characterizes the study sample overall and by subgroup. A total of 1031 T2DM subjects (of which 17.2% were female [n=177]) with an average age of 58.3 (standard deviation [SD]=9.8) years (range=40 to 86) were evaluated for this study. Note that while 1134 patients responded to the survey, only 1031 patients were analyzed because the remaining 103 patients had more than 50% missing responses on items.

### Study Survey

The survey contained a total of 30 questions assessing lifestyle, health status, and treatment preference (note: the 14 survey questions relevant to this report are provided in Appendix 1). The treatment preference questions were based on a modified version of the Health Belief Model (HBM)[20] and the Self-Regulation Model (SRM)[21] to target the rationale behind reporting that subjects "would like to use a QW treatment" as a function of adherence, barriers to treatment, and self-efficacy. The HBM suggests that beliefs about one's health condition, perceived benefits and barriers of treatment, and self-efficacy (belief in one's own ability to complete tasks and reach goals) explain adherence. The SRM is similar in that it suggests that beliefs about one's disease (cause, consequences, chronicity, etc.) and one's medication (necessity, side effect concerns, etc.) help to explain adherence. The modified survey addressed perceived disease characteristics (severity, acute versus chronic, controllability) and perceived susceptibility to diabetic complications as predictors of the perceived outcomes that survey respondents (i.e., participants who complete the survey) believed they would receive. These perceived benefits, along with perceived side effects and subject characteristics (such as number of medications they are already taking) are expected to influence a patient's adherence. The key variables collected through the survey and relevant here include:

- Subject characteristics: Demographics (age, sex, working status), current prescribed medication, lifestyle; and
- T2DM treatment preference: Perceived T2DM characteristics, adherence and barriers, self-efficacy, perception of QW.

**Table 2.** Overall and Group Demographic Characteristics of T2DM Patients (N=1031)

| Item | Overall (N=1031) | Oral Drugs Only (N=515) | Oral Drugs and Insulin (N=258) | Treatment naïve (N=258) | Chi-Square/ ANOVA | p-value* |
|---|---|---|---|---|---|---|
| **Gender** | | | | | | |
| Male | 854 (82.8%) | 453 (88.0%) | 217 (84.1%) | 184 (71.3%) | 33.8764 | <0.0001 |
| Female | 177 (17.2%) | 62 (12.0%) | 41 (15.9%) | 74 (28.7%) | | |
| **Age** | | | | | | |
| N | 1031 | 515 | 258 | 258 | 8.88 | 0.0001 |
| Median | 58.0 | 59.0 | 58.0 | 54.0 | | |
| Mean (SD) | 58.3 (9.8) | 59.4 (9.6) | 58.2 (9.4) | 56.3 (10.4) | | |
| Minimum – Maximum | 40.0 - 86.0 | 40.0 - 85.0 | 40.0 - 84.0 | 40.0 - 86.0 | | |
| **Full time/Part-time/Not working** | | | | | | |
| Full-time | 496 (48.1%) | 236 (45.8%) | 117 (45.3%) | 143 (55.4%) | 7.8618 | 0.0968 |
| Part-time | 104 (10.1%) | 53 (10.3%) | 26 (10.1%) | 25 (9.7%) | | |
| Not working | 431 (41.8%) | 226 (43.9%) | 115 (44.6%) | 90 (34.9%) | | |
| **Derived number of pills taken per day** | | | | | | |
| Less than 1 pill | 169 (16.4%) | 8 (1.6%) | 7 (2.7%) | 154 (59.7%) | 500.3173 | <0.0001 |
| 1 pill | 125 (12.1%) | 79 (15.3%) | 26 (10.1%) | 20 (7.8%) | | |
| 2 pills | 128 (12.4%) | 76 (14.8%) | 36 (14.0%) | 16 (6.2%) | | |
| 3 pills | 121 (11.7%) | 74 (14.4%) | 33 (12.8%) | 14 (5.4%) | | |
| 4 pills | 87 (8.4%) | 54 (10.5%) | 22 (8.5%) | 11 (4.3%) | | |
| 5 pills | 97 (9.4%) | 64 (12.4%) | 21 (8.1%) | 12 (4.7%) | | |
| 6 pills | 70 (6.8%) | 35 (6.8%) | 25 (9.7%) | 10 (3.9%) | | |
| 7 pills | 44 (4.3%) | 26 (5.0%) | 14 (5.4%) | 4 (1.6%) | | |
| 8 pills | 40 (3.9%) | 23 (4.5%) | 16 (6.2%) | 1 (0.4%) | | |
| 9 pills | 26 (2.5%) | 19 (3.7%) | 6 (2.3%) | 1 (0.4%) | | |
| 10 pills or more | 124 (12.0%) | 57 (11.1%) | 52 (20.2%) | 15 (5.8%) | | |

ANOVA: analysis of variance

*P-value of <0.05 implies statistically significant association between the demographic type and the treatment type.

### Statistical Methods

Using SAS version 9.4, a pre-screening logistic regression analysis was conducted using patients' T2DM formulation preference (e.g., QW administration) as the dependent variable and the key background variables (age, sex, working status, number of pills consumed) as the predictor variables to identify survey items that were predictive of dosing preference. The purpose of this was to parse out the variables (such as age, employment status, lifestyle variables, medications) that are predictive of the patient's treatment preference ($p \leq 0.10$). Patient's treatment preferences were measured using two survey items:

- "If you could be on any oral treatment regimen for your diabetes, how much are you willing to take the following treatment regimen?" For this item, respondents were presented with four treatment regimens (once a week, once a day, twice a day, and three times a day) and rated each as "Won't take it," "Probably won't take it", "Not sure", "Probably will take it," and "Will take it."
- "If you could be on any oral treatment regimen for diabetes, which would you prefer?" For this item, respondents were provided with four response options including once a week, once a day, twice a day, and three times a day.

The predictive variables were subsequently used as the basis for partitioning in classification and regression (CART) decision trees. CART analysis was conducted in R Gui using the package "rpart" and "tree."[22]

Following the identification of key variables predictive of dosing preferences, frequencies were reported for patient characteristics by dosing preference. Specifically, (1) frequency-of-dosing preference was reported by current treatment group (i.e., OAD, OAD+insulin, treatment naïve), demographic groups (e.g., age), current dosing regimen, lifestyle (e.g., work status), other prescribed medication, and (2) subjects' understanding of the importance of diet and exercise by subjects' dosing preference (i.e., QW and QD), and (3) frequency for reason for QW preference reported overall.

## RESULTS

Preliminary logistic regression and CART analysis revealed current treatment regimen, age, and work status as the major demographic factors most predictive of QW preference. As shown in Figure 1, the QD regimen is consistently preferred across the groups (45% – 62%), with groups 1 and 2 having the highest proportion (62% and 53.8%, respectively) of patients endorsing QD regimen and 42% of patients in the treatment naïve group endorsing QD regimen. The second most preferred dosing regimen was QW; overall, 33.3% of patients preferred QW rather than other QD, BID, or TID. The treatment naïve patient group (n=258) had the highest comparative preference towards QW: 45.7% compared to OAD (27.8%) and OAD+Insulin (31.8%). Further, the number of patients preferring the QD regimen tends to increase (Figure 2) in elderly populations (e.g., ≥65 years old versus ≤64 years old) while, in contrast, the preference for QW regimen tends to increase in younger populations (e.g. age ≤64 versus age ≥65). Additionally, the preference for QD regimen tends to also increase in patients taking 2 – 5 drugs, whereas the preference for QW regimen is higher in patients taking one drug or fewer, or 6 or more drugs (Figure 3).

In particular, the majority of younger (≤64 years), full-time-working subjects who are treatment naïve and do not have comorbidities (n=83) report a preference (69%) for QW dosing relative to the ones who show preference for QD regimen (30%) (Figure 4).

Overall, the most commonly cited reasons for QW preference were (1) "less burdensome because they didn't have to take it every day" (47.8%), (2) "less psychological burden" (14.6%), and (3) "forget to take it less often"(12.5%). There were no significant associations between subject understanding of importance of diet and exercise on blood glucose levels and dosing choices (p>0.05). The same was also true for the subgroup of patients who were young, not on medication, working full time, and took no medications for comorbid conditions. Figures 5-6 reveal no significant differences in treatment naive patients' perceptions of diet or exercise affecting their glucose level based on their dosing preferences. Approximately 32% and 28% of patients who chose weekly dosing (QW) reported "somewhat true" on physical activity and diet, respectively, influencing their glucose level. Among patients choosing daily dosing (QD), 42% and 37% patients reported "somewhat true" perceptions about physical activity and diet, respectively, impacting their glucose level. For the response category "very true," approximately 51% and 53% of patients who chose QW endorsed "very true" on physical activity and diet, respectively, influencing their glucose level. Forty-two percent and 45% of patients who chose daily dosing endorsed "very true" on physical activity and diet, respectively, influencing their glucose level. Similar to the treatment naive group, patients in the overall population showed similar trends, wherein, no significant differences were found in patients' perceptions of diet or exercise affecting their glucose levels based on their dosing preferences.

**Figure 1.** Frequency of Dosing Preference by Groups 1-3 and Overall

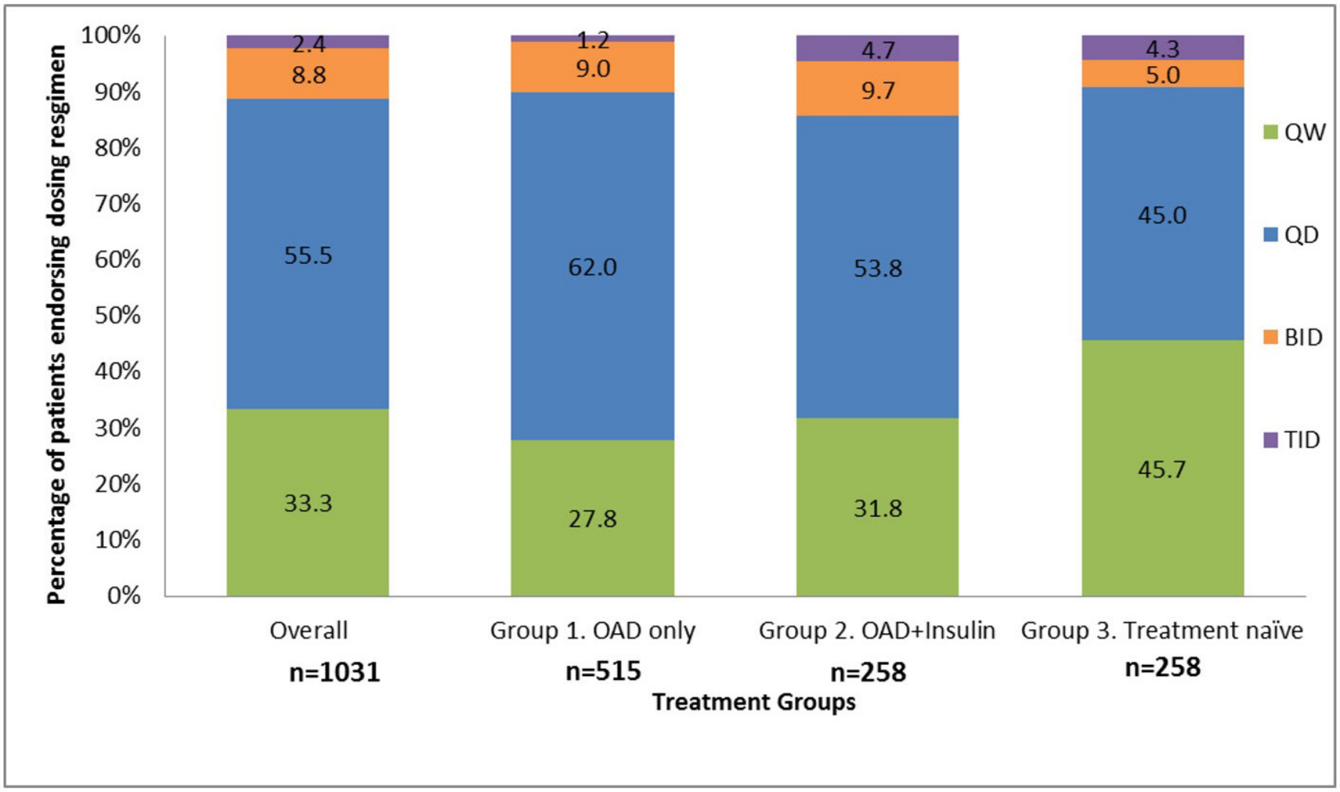

TID: Thrice a day, BID: Twice a day, QD: Once daily, QW: Once weekly

**Figure 2.** Frequency of Dosing Preference by Age Groups

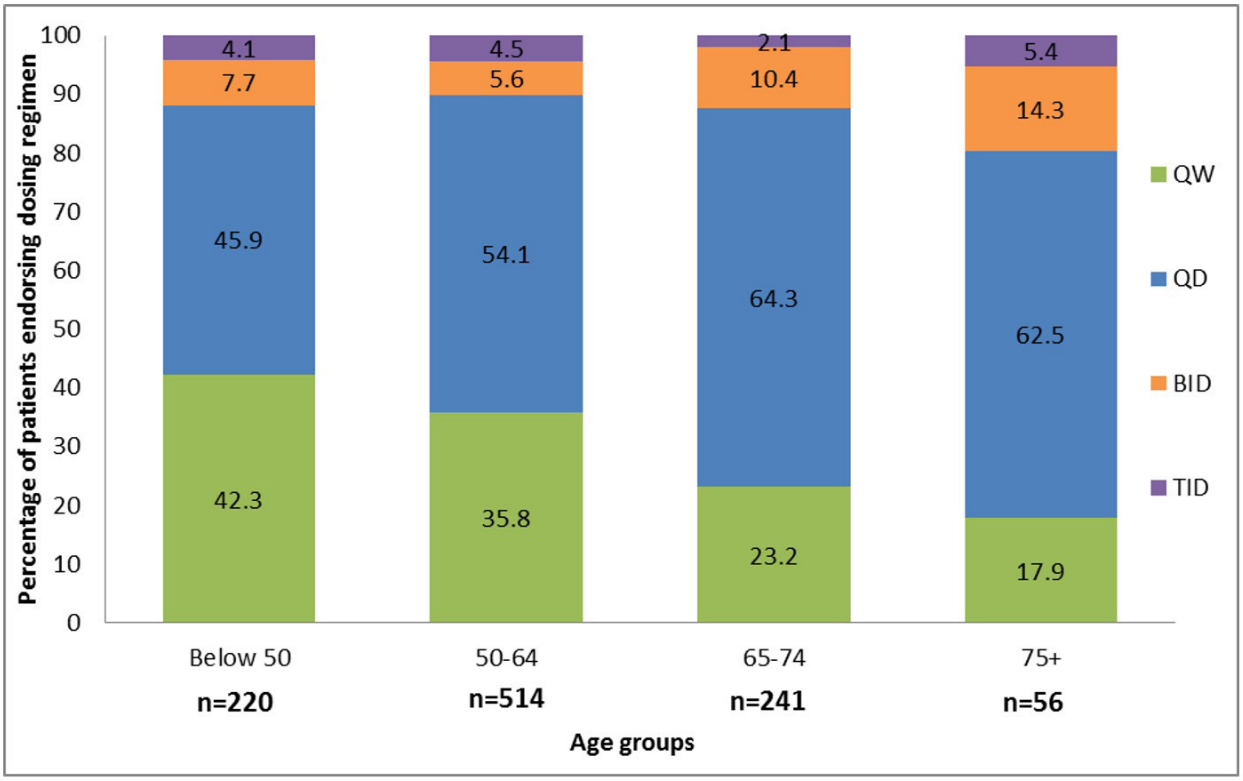

TID: Thrice a day, BID: Twice a day, QD: Once daily, QW: Once weekly

**Figure 3.** Frequency of Dosing Preference by Number of Pills Consumed

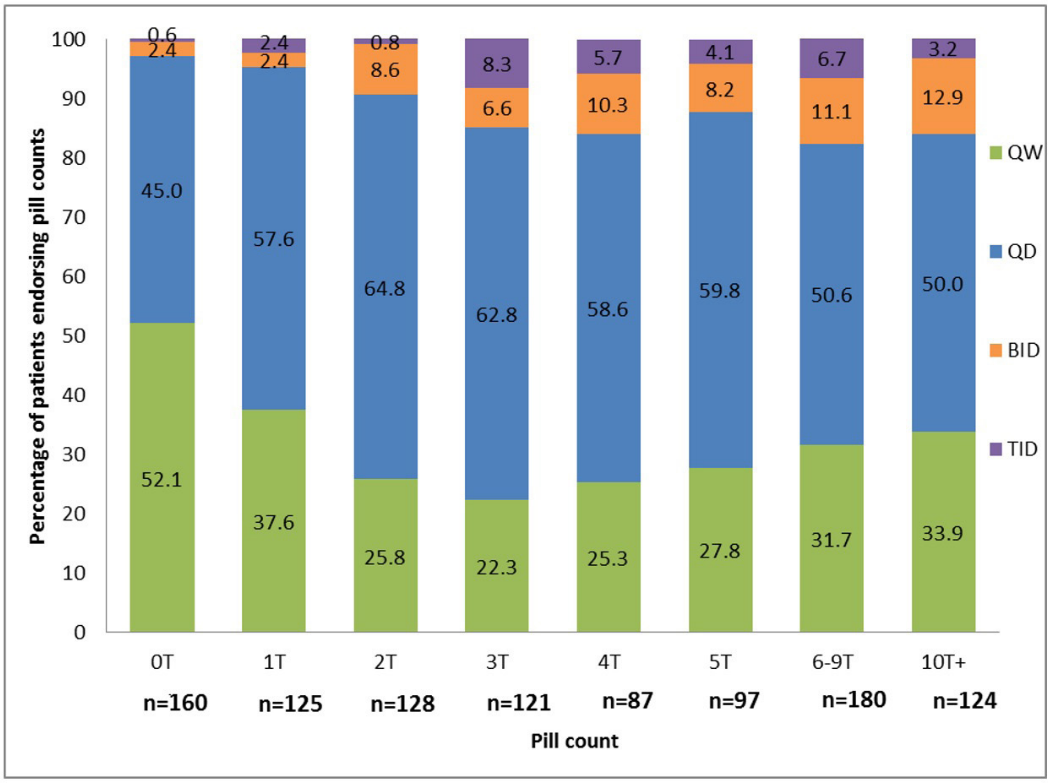

TID: Thrice a day, BID: Twice a day, QD: Once daily, QW: Once weekly

**Figure 4.** Dosing Preferences of Treatment Naive Young, Full-time Working Patients with T2DM who are not on any medications including T2DM drugs

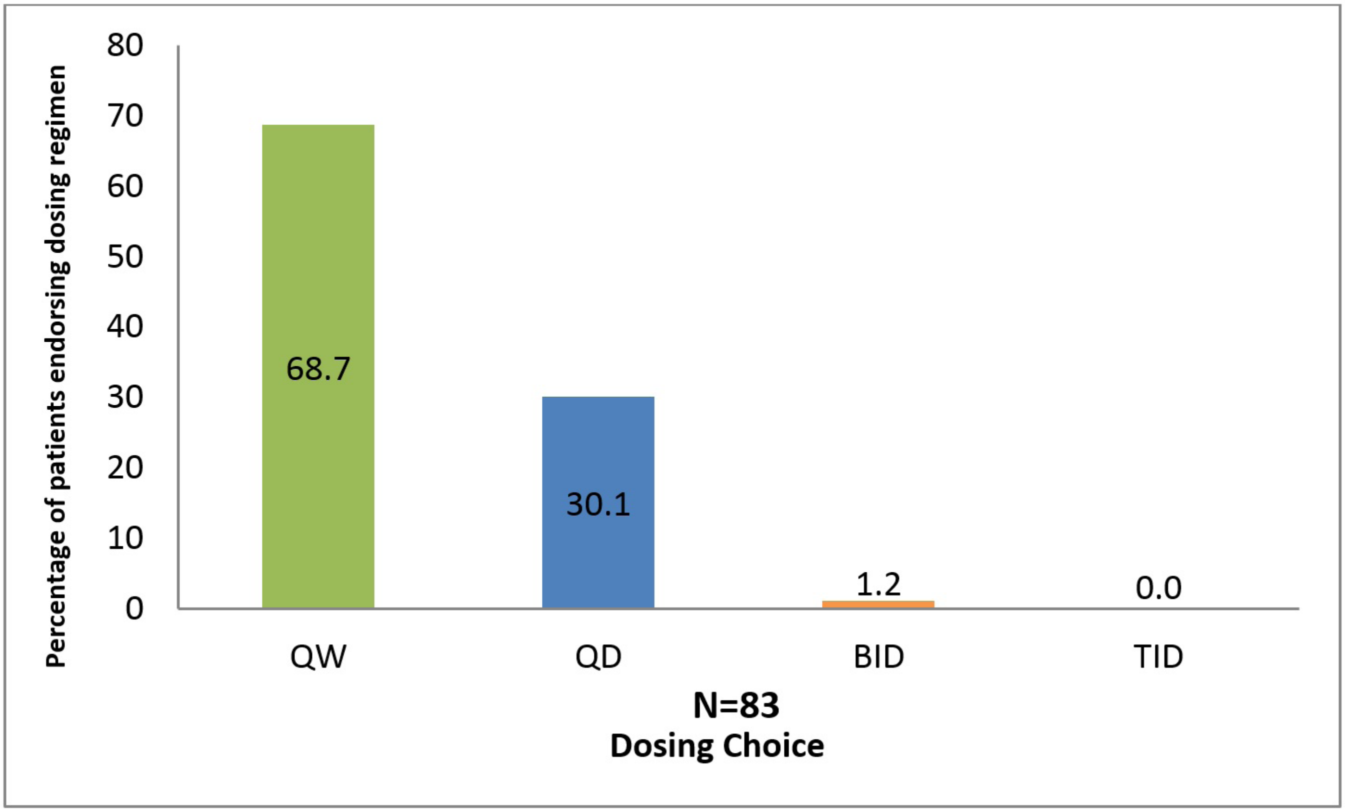

TID: Thrice a day, BID: Twice a day, QD: Once daily, QW: Once weekly

**Figure 5.** Dosing Preferences for Treatment Naive Groups of Patients with different Perceptions of Physical Activity influencing their Glucose Level

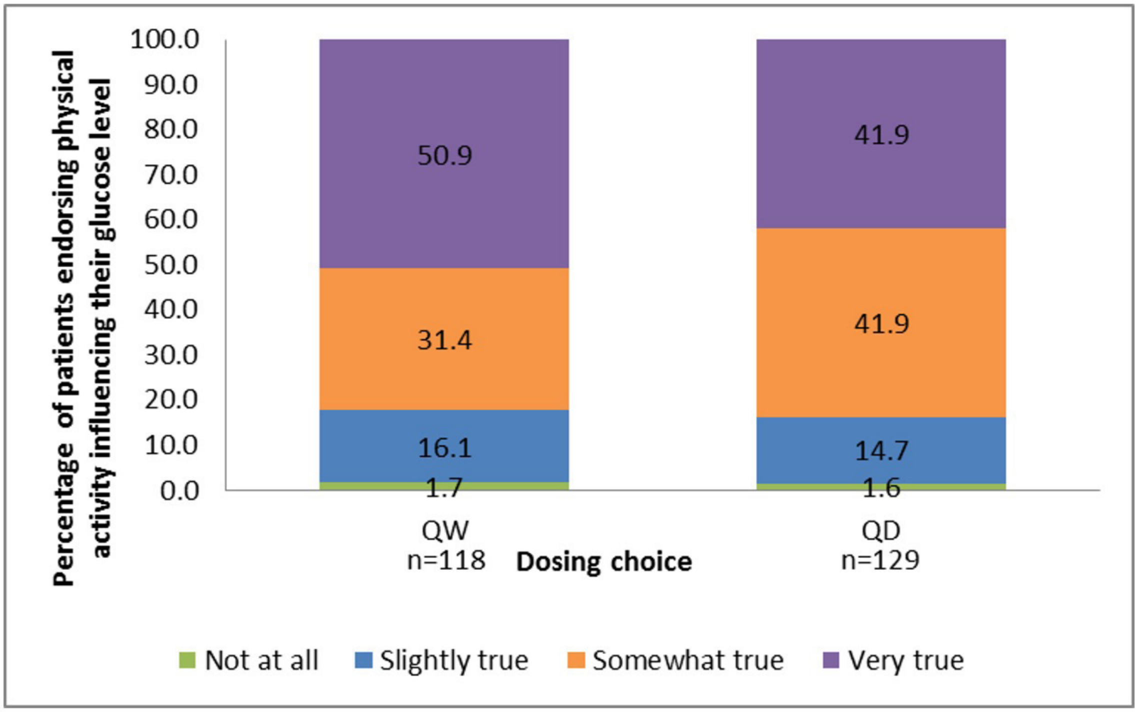

QD: Once daily, QW: Once weekly

**Figure 6.** Dosing Preferences for Treatment Naive Groups of Patients with different Perceptions of Food (diet) Influencing their Glucose Level

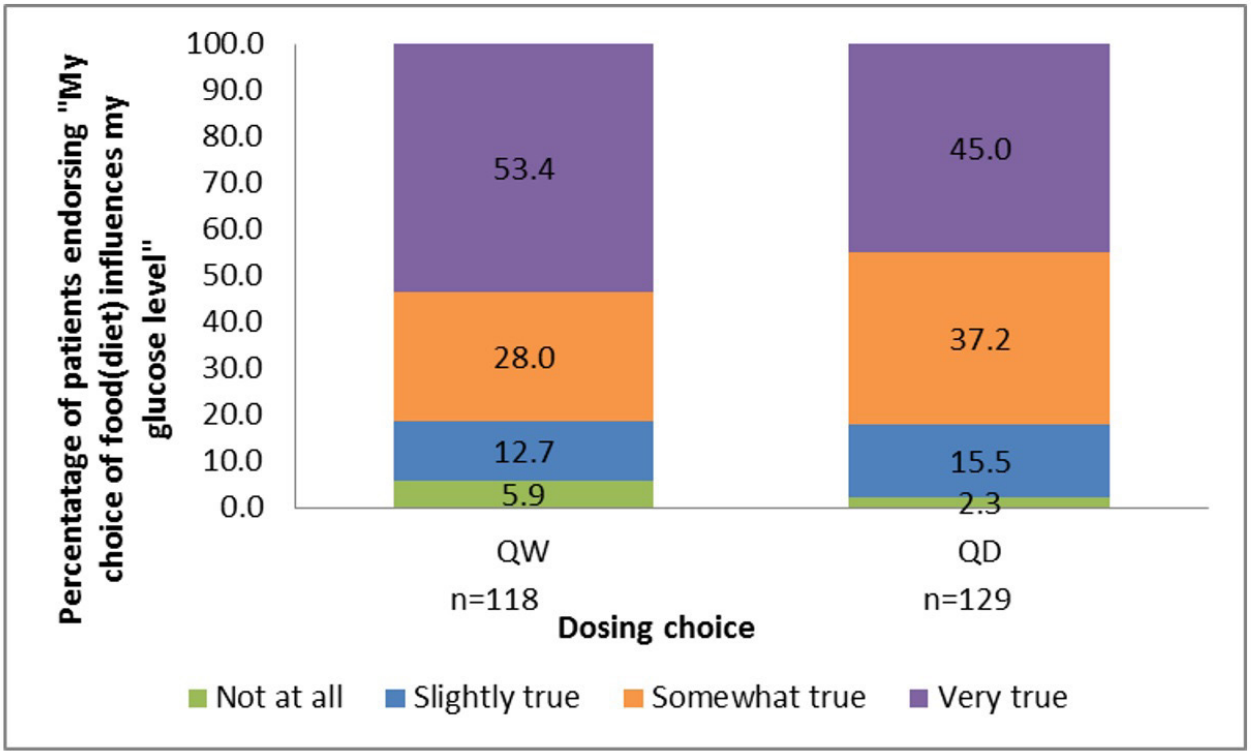

QD: Once daily, QW: Once weekly

## DISCUSSION

In addition to regimen complexity, adherence is influenced by patient preferences and priorities,[23] as well as beliefs about health, disease, and medication,[20,21,24] and to account for these factors, the American Diabetes Association and European Association for the Study of Diabetes has recommended taking a patient-centered approach to the management of T2DM.[25] Thus, it is important to determine what schedule patients may prefer and are therefore more likely to adhere to. The present data suggest that, overall, QW medication was preferred across a wide range of patient characteristics, primarily in the younger population (≤64 years). This finding is consistent with existing research that explores correlations between patient dosing preferences, adherence, and dosing burden; for example, a recent study by Hauber et al., indicated that younger patients tend to prefer once-weekly dosing of antihyperglycemic treatment, especially those patients who are not currently on treatment (treatment naïve).[26] In the current sample of 83 treatment naive patients, the majority (n=57 [69%]) of young, full-time-working patients with T2DM who are not on any medication prefer once a week treatment regimen. Furthermore, there is no difference in understanding of exercise or diet's impact on glucose levels by dosing choice. Young, treatment naïve, full time working patients tend to prefer QW primary because they find it less burdensome that they don't have to take pills every day and feel that they would forget to take it less often. In addition, preference for QW treatment also increases in patients who had to take 0-1 pill or 6 pills or more, with the primary reason again being that it was less burdensome to do so and also most convenient to take large number of pills QW while traveling.

This study is not without limitations. First, less than 20% of the study population was female. Though studies show that men have a higher prevalence of DM than women across a variety of Asian subgroups[27-29] and the Japanese population and the large sample size in the present study may overcome gender oversampling, additional research may be warranted to confirm gender-based dosing preferences. Additionally, the online survey format may have resulted in recruitment of a younger sample than the general T2DM population (i.e., subjects who may be more familiar or comfortable with participating in web-based activities) though analysis of demographic variables indicated that a wide variety of age groups participated (range = 40 to 86 years with an average age of about 58 years). Next, the study design relied upon the veracity of the potential subject's self-report to identify them as either T1DM or T2DM. While certain types of self-report may be prone to bias,[30] however, other research suggests that patients can accurately self-report their chronic health conditions and such data can even be reasonably relied upon for broad measures of population-level disease prevalence rates.[31] Finally, there was no QW oral T2DM regimen available at the time of the study, and subjects may have had difficulty comparing such a theoretical regimen to the QD regimen with which most were familiar. As QW regimens become increasingly available, additional research may be needed to demonstrate actual preference rates as opposed to the expected preference rates presented here.

## CONCLUSION

Consistent with previous research and best practices, results presented here support the importance of taking a patient-centered approach to the management of T2DM, as, when given a choice, patients with the condition have varying preferences in their dosing regimens. With a greater number of treatment options available, including the availability of QW dosing, it is important to understand the characteristics of patients who prefer a specific dosing regimen to maximize the probability of adherence and, ultimately, improved health outcomes. In this regard, there was a tendency for QW dosing to be preferred by subjects who were treatment naïve (i.e., patients diagnosed with T2DM and on diet/exercise therapy with no current or past experience with drug treatment for T2DM), younger (age <65 years), working full-time, and/or currently taking 0 or 1 drugs or more than 6 drugs). Among the most important reasons that subjects reported a preference for a QW dosing regimen included it being less burdensome, being more convenient when traveling, and having less psychological burden, reasons that are justifiable for busy, young, patients working full-time, who have limited mental bandwidth to consistently remember taking higher dosing frequency medications. With this evidence, physicians may be better prepared to understand who among their patients will be better suited for a QW dosing regimen and, thus, to tailor their treatment to the individual patient.

## CONFLICT OF INTEREST DECLARATION

Financial sponsors: MSD K.K. a subsidiary of Merck & Co., Inc., Kenilworth, N.J., U.S.A. Drs. Sen and Sheilds report personal fees from MSD K.K during the conduct of the study. Dr. Atsuda reports grants from MSD K.K. during the conduct of the study; personal fees from MSD K.K., personal fees from TAKEDA K.K., personal fees from ASTELLAS K.K., personal fees from DAIICHI SANKYO K.K., and personal fees from MITSUBISHI TANABE K.K. outside the submitted work.

## Figures and Tables

**Figure:** Supplementary Content

